# Two variants among *Haemophilus influenzae *serotype b strains with distinct *bcs4*, *hcsA *and *hcsB *genes display differences in expression of the polysaccharide capsule

**DOI:** 10.1186/1471-2180-8-35

**Published:** 2008-02-25

**Authors:** Leo Schouls, Han van der Heide, Sandra Witteveen, Bert Zomer, Arie van der Ende, Marina Burger, Corrie Schot

**Affiliations:** 1 Laboratory for Infectious Diseases and Perinatal screening, National Institute for Public Health and the Environment, Antonie van Leeuwenhoeklaan 9, 3721 MA Bilthoven, The Netherlands; 2 Netherlands Vaccine Institute, Antonie van Leeuwenhoeklaan, Bilthoven, The Netherlands; 3 Netherlands Reference Laboratory for Bacterial Meningitis, Department of Medical Microbiology, Academic Medical Centre, Amsterdam, The Netherlands; 4 Laboratory for Toxicology, National Institute for Public Health and the Environment, Antonie van Leeuwenhoeklaan, Bilthoven, The Netherlands

## Abstract

**Background:**

Despite nearly complete vaccine coverage, a small number of fully vaccinated children in the Netherlands have experienced invasive disease caused by *Haemophilus influenzae *serotype b (Hib). This increase started in 2002, nine years after the introduction of nationwide vaccination in the Netherlands. The capsular polysaccharide of Hib is used as a conjugate vaccine to protect against Hib disease. To evaluate the possible rise of escape variants, explaining the increased number of vaccine failures we analyzed the composition of the capsular genes and the expressed polysaccharide of Dutch Hib strains collected before and after the introduction of Hib vaccination.

**Results:**

The DNA sequences of the complete capsular gene clusters of 9 Dutch Hib strains were assessed and two variants, designated type I and type II were found. The two variants displayed considerable sequence divergence in the *hcsA *and *hcsB *genes, involved in transport of capsular polysaccharide to the cell surface. Application of *hcsA *type specific PCRs on 670 Hib strains collected from Dutch patients with invasive Hib disease showed that 5% of the strains collected before 1996 were type II. No endogenous type II Hib strains were isolated after 1995 and all type II strains were isolated from 0–4 year old, non-vaccinated children only. Analysis of a worldwide collection of Hib strains from the pre-vaccination era revealed considerable geographic differences in the distribution of the type I and type II strains with up to 73% of type II strains in the USA. NMR analysis of type I and type II capsule polysaccharides did not reveal structural differences. However, type I strains were shown to produce twice as much surface bound capsular polysaccharide.

**Conclusion:**

Type II strains were only isolated during the pre-vaccination era from young, non-vaccinated individuals and displayed a lower expression of capsular polysaccharide than type I strains. The higher polysaccharide expression may have provided a selective advantage for type I strains resulting in the rapid elimination of type II from the Dutch Hib population after introduction of nationwide Hib vaccination. However, this phenomenon does not explain the increase in the number of Hib vaccine failures in the Netherlands.

## Background

Worldwide the number of cases of invasive disease caused by *Haemophilus influenzae *serotype b (Hib) has dramatically decreased after the introduction of vaccines composed of the conjugated polysaccharide capsule of the pathogen. In the Netherlands Hib vaccination was introduced in 1993 resulting in a decrease in incidence of invasive Hib disease among children younger than 5 years of age from 28.7 per 100,000 in 1992 to 0.4 in 2001 [1].

Despite nearly complete vaccine coverage, a small number of fully vaccinated children in the Netherlands have experienced invasive Hib disease. In 2002 the incidence of vaccine failures in Dutch children aged 0–4 years increased from 0.4 per 100,000 in the years 1996 to 2001 to 1.3 per 100,000 in 2002 [2,1]. In only a few patients underlying conditions were found that might have contributed to vaccine failure. Half of the patients had adequate anti-PRP titers at the onset of disease, which is similar to what is found in the same age group of the healthy population. All vaccine failure patients responded well to revaccination with the Hib vaccine yielding high anti-PRP titers, indicating there was no impairment of the immune system. Since 2002 the annual number of cases of invasive Hib disease among 0–4 year old children in the Netherlands has remained approximately the same [3].

In the United Kingdom a somewhat more pronounced rise in vaccine failures has been observed particularly in children 1–4 years of age and this was believed to be caused by inadequate antibody titers against Hib due to waning immunity. To compensate for the reduced antibody titers, a catch-up campaign, designed to boost immunity in children aged 6 months to 4 years of age, was implemented in the United Kingdom [4]. Furthermore, an additional Hib booster in the second year of life has now been implemented in the United Kingdom vaccination schedule [5]. A Hib booster vaccination at 11 months of age has been part of the Dutch Hib vaccination schedule since its introduction in 1993. This booster should have prevented waning immunity in children and suggests that reduced antibody titers may not have been the cause of the increased number of vaccine failures observed in the Netherlands. Therefore, changes in the properties, e.g. virulence factors, of circulating Hib strains could not excluded. The major virulence factor of Hib is the polysaccharide capsule, a polymer of ribose ribitol phosphate (PRP), which is also the antigen used for Hib vaccination. The production and export of the polysaccharide capsule of Hib is encoded by 10 genes located in single locus consisting of 3 distinct regions. The first region contains 4 *bex *genes involved in the export of the capsular polysaccharide, the second region carries 4 *bcs *genes which are considered to be involved in the actual synthesis of the polysaccharide and the third region carrying 2 *hcs *genes is now also considered to be involved in the export of the polysaccharide to the surface of the cell [6,7].

Changes of the genes in the capsular gene cluster locus may lead to an altered capsule of the Hib. If this would be the case in the currently circulating Hib strains the vaccine induced immunity may not provide optimal protection causing an increased incidence of invasive Hib disease among vaccinated children in the Netherlands. This prompted us to analyze the composition of the genes encoding for capsular polysaccharide, the structure of the encoded polysaccharide and of the level of expressed capsular polysaccharide in Hib strains isolated before and after the introduction of nationwide Hib vaccination in the Netherlands.

## Results

### Sequence analysis of the Hib capsule locus

**Figure 1:**
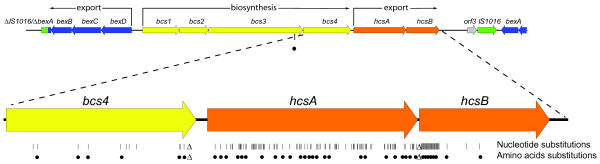
**Schematic representation of the differences between the type I and type II Hib capsular gene clusters**. The top part of the figure shows the genetic organization of the capsular gene cluster of Hib. The figure displays a single copy of the gene cluster with the fused truncated insertion element IS1016 and the incomplete *bexA *gene on the left hand side. The complete *bexA *gene and part of the *bexB *gene, present in the second copy of the gene cluster are depicted at the right hand side. The region containing the *bcs4*, *hcsA *and *hcsB *genes are displayed as a blow up. The vertical lines indicate nucleotide substitutions, closed circles denote amino acid substitutions and Δ indicate deletions.

The DNA sequence of the locus carrying the genes encoding the components required to synthesize and export the capsular polysaccharide was determined. Sequencing of the complete capsular gene cluster was performed on 9 Hib strains isolated from Dutch patients with invasive disease, on reference strain Eagan and on strain 1482 which has been used for the production of the Hib conjugate vaccine (Table 1). The 9 clinical isolates were selected because they represented pre- and post-vaccination strains and because they were isolated from vaccinated and non-vaccinated patients of various ages. Analysis revealed that the DNA sequence of the complete 17 kb capsule gene cluster was identical among 8 of the 9 Dutch Hib strains. The gene cluster of the divergent Hib strain, isolated from an 18 months old non-vaccinated patient in 1994, differed considerably from that of the other 8 strains (Fig. 1). We designated strains with the composition of the gene cluster found in the 8 strains as Hib type I and the strain with the aberrant gene cluster as Hib type II. Sequence analysis of the gene cluster of the Eagan strain and of the Hib vaccine strain 1482, yielded sequences that were virtually identical to the Dutch type II strain, with the exception of a single, silent C→T mutation at position 194 of the *hcsA *gene. The differences between the DNA sequences of the type I and type II capsule gene clusters were restricted to *bcs3 *(a single nucleotide polymorphism (SNP)), the *bcs4 *(12 SNPs + 24 bp deletion in type II), *hcsA *(93 SNPs) and *hcsB *(24 SNPs + 24 bp deletion in type II) genes. The 24 bp deletion in the *bcs4 *gene of type II strains results in an 8 amino acids loss in the C-terminal part of the product encoded by this gene. The 24 bp deletion of the *hcsB *gene was caused by a 2 bp deletion in the intergenic region between *hcsA *and *hcsB*, resulting in the loss of the ATG start codon of *hcsB*. However, a C→G SNP created a new start codon in the now 24 bp shorter *hcsB *gene. As a result HcsB in type II strains have an 8 amino acid shorter signal sequence.

**Table 1:** Strains used for sequencing the capsule gene cluster

Strain number	Age patient	Isolated from	Vaccine failure	Capsule locus type
Hib from pre-vaccination era				
H1990-0614	14 months	CSF	No	I
H1991-1722	3 years	CSF	No	I
H1992-2218	10 months	CSF	No	I
H1994-0151	18 months	CSF	No	II
Hib from post-vaccination era				
H2000-1692	71 years	Blood	No	I
H2000-1882	2 years	CSF	Yes	I
H2001-1610	3 years	Blood	Yes	I
H2002-2067	8 months	CSF	Yes	I
H2002-2328	73 years	Blood	No	I
Other Hib				
Vaccine strain 1482			No	II
Strain Eagan			No	II

Comparison with the sequences in the publicly available databases revealed that sequences of the capsule gene clusters from 3 different Hib strains have been published [6]. Although the sequences were not identical, they were highly similar (e.g. 17 bp difference between type II sequences DQ368335 and AF54213) and it was clear that the capsule gene cluster in one of these 3 strains, a Hib minus strain isolated from a patient in the USA, is of type I (acc.no. AF549212). The capsule gene clusters of the other 2 strains, a regular Hib strain and a Hib minus strain, have a type II composition (acc.no. AF549213 and AF549210). Currently, the Sanger Institute, in collaboration with Dr. Derrick Crook and Prof. Richard Moxon, is sequencing the genome of a Hib isolate (strain 10810) from a meningitis patient from the United Kingdom [8]. The nucleotide sequence of the capsular genes in the unfinished genome sequence of this strain 10810 is identical to that of the type I capsular gene cluster found in the Dutch strains.

### Distribution of Hib type I and type II strains

The availability of the DNA sequence of the 2 types of capsular gene clusters enabled us to design 2 primer sets targeting *hcsA *that could discriminate between the two capsular genotypes in a PCR. This PCR was used to analyze 670 Hib isolates obtained from patients with invasive Hib disease in the Netherlands. Of 388 strains isolated during the period 1983–1995 and of 282 strains isolated during the period 1996–2006, a type II associated *hcsA *gene was found in 20 (5%) and 1 (0.4%) isolates, respectively (Table 2), yielding a statistically highly significant difference in distribution (χ^2^, p ≤ 0.001). Remarkably, with the exception of a single Hib strain isolated in 2005, type II strains were not detected among the Hib strains isolated after 1995, which is only 2 years after the introduction of the Hib vaccine in the national vaccination program. The type II Hib strain isolated in 2005 was isolated from a 3 months old, non-vaccinated Chinese child that was adopted by Dutch parents and did not represent an endogenous strain. All type II strains were isolated from non-vaccinated children ranging from 0–4 years of age, not a single type II strain was isolated from a patient older than 4 years of age. Again this difference in distribution is statistically significant (χ^2^, p ≤ 0.001). There was no statistically significant association between the distribution of the capsular genotypes and the materials from which they were isolated (CSF or blood).

**Table 2:** Distribution of the type I and type II strains stratified over time and age groups

			Capsule locus type	
Time period of isolation	Patient's age	No. of strains	I	II

1983–1995	All	388	368	20
	≤ 4 years	255	235	20
	> 4 years	133	133	0
1996–2006	All	282	281	1
	≤ 4 years	143	142	1
	> 4 years	139	139	0

Assessment by the type I and type II specific PCRs of a collection of Hib strains isolated during the pre-vaccination period between 1980 and 1990 from patients in various regions in the world revealed remarkable differences in geographic distribution of type I and type II (Table 3). Of the 232 Dutch Hib isolates from this time period 11 were type II (5%). None of the 17 Hib isolates from patients from other parts of Europe carried the type II *hcsA *gene. The proportion of the type II strains among the strains from the various global regions varied from 47% for strains from the African continent, 4% for Asian strains, 8% in strains from Australia and 43% of the isolates from Cuba. However, the largest proportion of type II strains was found among Hib strains isolated from patients in the United States where 40 of the 55 strains were type II (73%).

**Table 3:** Distribution of capsular genotypes in various geographic regions among Hib strains collected during the pre-vaccination era 1980–1990

		Capsule locus type		
		
Region	No. of strains	I	II	% II
The Netherlands	232	221	11	5
Europe (Finland & Switzerland)	17	16	0	0
Africa (The Gambia & South Africa)	17	9	8	47
Asia (Malaysia & Vietnam)	26	25	1	4
Australia	12	11	1	8
Cuba	7	4	3	43
USA	55	15	40	73

### Sequence variation of the hcsA gene

We found only 2 variants of the capsule gene cluster in the 11 strains from which the complete gene cluster was sequenced, suggesting very limited variation of the capsular genes. To verify this finding we determined the DNA sequence of *hcsA *PCR products obtained from a large number of type I and type II strains. In total the DNA sequences of the *hcsA *PCR products obtained from 111 Dutch type I strains collected during the time period 1984 to 2006 were determined. The DNA sequences of 109 of the 111 PCR products were identical. Only two strains, one isolated in 1992 from a 99 year old patient and the other in 1996 from 4 months non-vaccinated old child, had an A→G SNP at position 597 of the type I *hcsA *gene that did not lead to an amino acid substitution. The DNA sequences of the *hcsA *PCR products from all 21 type II strains in the strain collection were determined and all products had identical sequences. In addition, the sequences of the *hcsA *PCR products of 14 type I and 13 type II Hib strains collected during the pre-vaccination era in various other parts of the world were also determined. This revealed very limited variation in the *hcsA *sequence of type I strains. As, The A→G SNP at position 597 of the type I *hcsA *gene which was also found in the Dutch Hib strains, appeared to be present in a strain from South-Africa, in one from Malaysia, one from Vietnam and in 2 strains from the USA. Furthermore, 2 strains from Cuba contained 2 SNPs: an A→G SNP at position 736 and a C→A SNP at position 806. Both base pair changes lead to amino acid substitutions. Remarkably, these SNPs correspond to the sequences found in the type II *hcsA *gene. All PCR products obtained from the 13 type II strains had sequences identical to the previously determined type II *hcsA *sequence.

### Association between capsular type and genotype

**Figure 2:**
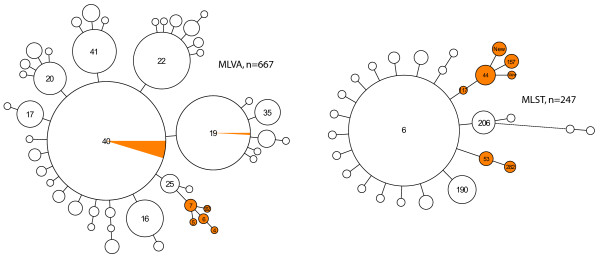
**Minimum spanning trees of 667 strains on which MLVA was performed and of 247 Hib strains analyzed by MLST**. Each circle represents a different type. The size of the circle indicates the number of strains with this particular type. The orange area in the circles indicates the portion of type II strains. The numbers in the circles denote the MLST sequence type or MLVA type. Only the numbers of the predominant types and the types carrying capsular genotype II are displayed.

Assessing 667 of the Dutch Hib strains by MLVA [9], revealed that the type II strains were not randomly distributed among the 47 MLVA types. Of the 22 type II strains, 13 (59%) had MLVA type 40 which is the predominant type among Dutch strains, one strain belonged to MLVA type 19 and the remaining 8 strains formed a separate branch of MLVA types not found among type I strains (Fig. 2). Of the collection of Dutch strains available, 247 Hib strains were analyzed by MLST [10,9] and none of the 19 type II strains among this collection were of sequence type 6 (ST-6), which was by far the predominant sequence type (75%) among the 247 Hib strains (Fig 2). This suggested an inverse relationship between capsule locus type II and ST-6. To assess such a possible inverse relationship we amplified and subsequently sequenced the MLST *fucK *locus for all 89 type II Hib strains from our collection, including strains isolated in other countries. All strains with a *fucK *allele other than allele number 5 were classified as not having ST-6. Only a few type II Hib strains yielded *fucK *allele 5 and for these strains the MLST *pgi *locus was amplified and subsequently sequenced. None of these strains carried *pgi *allele 7. As *pgi *allele 7 and *fucK *allele 5 are part of the ST-6 allelic profile this showed that none of the type II isolates had ST-6, demonstrating an absolute inverse relationship between capsule locus type II and ST-6 genotype for the 89 type II Hib strains included in this study.

### Composition of the capsular polysaccharide obtained from type I and type II strains

**Figure 3:**
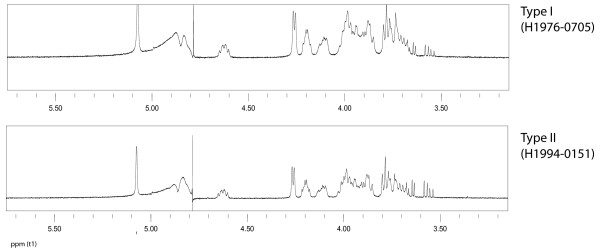
**The 1H-NMR spectra of PRP samples obtained from type II and type I Hib strains**. NMR profiles of type I Hib strain H1990-0614 and type II Hib strain H1994-0151. The minor absorptions at 3.5–3.65 ppm are caused some residual glycerol.

The sequence differences between the homologous genes in the type I and type II capsule loci prompted us to analyze the structure of capsular polysaccharides produced by type I and type II strains. NMR analysis of polysaccharides isolated from the culture supernatant of two type I and two type II strains showed no obvious differences between NMR spectra of the polysaccharides obtained from the two types, suggesting the structure of the polysaccharide from the two types is identical (Fig. 3). The few very minor differences in the NMR spectra were not associated with the two capsular genotypes.

### Level of expression of polysaccharide capsule in type I and type II Hib strains

**Figure 4:**
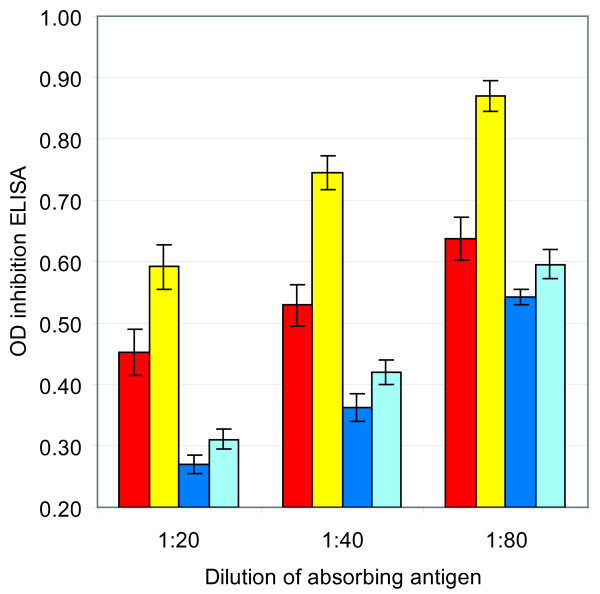
**Expression of the Hib polysaccharide capsule of type I and type II strains as measured by inhibition ELISA**. The graph displays the OD values obtained in the ELISA after inhibition by the cell fraction and the culture supernatant obtained from type I and type II strains. Inhibition was performed with 3 different dilutions of the antigens. Each bar denotes the average of the values obtained with 4 different strains and the error bars indicate the standard error. Red bars, inhibition by type I cells; yellow, inhibition by type II cells; dark blue, inhibition by type I culture supernatant; light blue, inhibition by type II culture supernatant. Note that in this graph higher OD values represent lower polysaccharide expression.

In order to detect differences in the level of capsule polysaccharide expression by capsule locus type I and type II strains, polysaccharide production was assessed by an inhibition ELISA. Four type I and 4 type II strains were analyzed to determine the level of polysaccharide production. The results showed that absorption of serum with cell fractions of the type I cultures caused more inhibition than with cells of type II strains. Using a standard curve based on inhibition with serial dilutions of purified polysaccharide, we deduced that the type I cells contained approximately twice as much capsular polysaccharide as type II cells (Fig. 4). This result was reproducible (the same experiment was performed three times) and statistically significant (ANOVA analysis, p ≤ 0.002). The culture supernatants from type I and type II cultures contained significantly more polysaccharide than the cellular fractions. However, the amount of polysaccharide in the culture supernatant from type I strains did not differ significantly from that of type II strains. This indicates that the amount of shed polysaccharide in type I and type II strains is the same, but that the total amount of polysaccharide produced in type I strains is higher.

**Figure 5:**
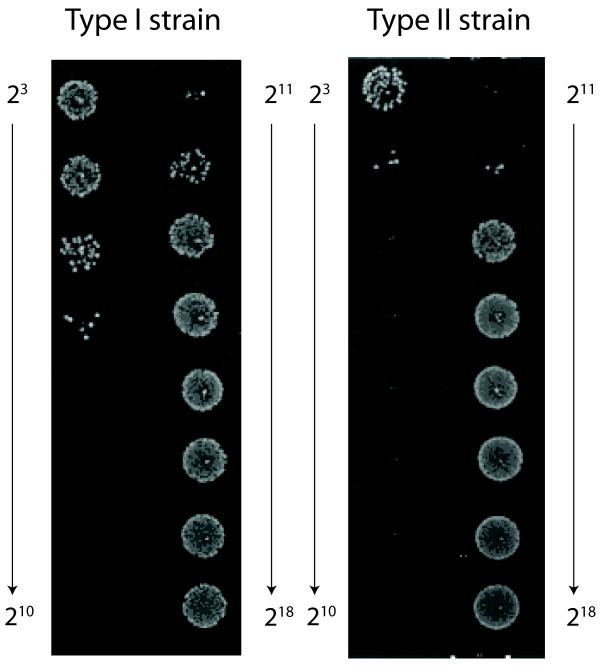
**SBA with serial dilutions of a high titer serum using type I and type II Hib strains as target cells**. The serum sample was diluted over a range of 1:8 (2^3^) to 1:262,144 (2^18^) and mixed with type I or with type II bacteria in the presence of baby rabbit complement. The SBA titer using the type I strain as target is 1:2,048 (2^11^), whereas the type II target strain yields a titer of 1:4,096 (2^12^). The pro-zone effect, growth in the presence of high concentrations of serum, is more pronounced for the type I strain.

Possible differences in the level of polysaccharide production were also assessed in a serum bactericidal assay. The SBA was performed using heat inactivated serum obtained from adult human volunteers that were immunized with Hib conjugate vaccine. Serial dilutions of the serum were used in SBAs using type I and type II Hib as target cells. Twice as much serum was required to kill 90% of the type I target cells in the SBA compared to the amount required to kill type II target cells (Fig. 5). Remarkably, the use of type I strains as target cells yielded a more pronounced pro-zone effect than type II strains. Cell fractions of the 4 type I and 4 type II strains used in the above described inhibition ELISA were used to perform absorption of a high titer serum from a human volunteer. Absorption of the serum with cells from the type I strains reduced its SBA titer from 1:2,048 to 1:32. In contrast, the serum sample retained an SBA titer of 1:128 after absorption with type II cells (Table 4.). The same 4-fold difference in reduction of SBA titer was observed if either type I or a type II Hib strains were used as target cells for the SBA. Other high titer serum samples absorbed with type I cells yielded similar results. Absorption with cell fractions of isogenic type I or type II Hib minus variants did not reduce the SBA titer, showing the observed reduction is caused by the polysaccharide and not by other components of the bacteria.

**Table 4:** Reduction in SBA of a high titer serum sample absorbed with type I or type II Hib cells

	SBA titer	
	
	Type I target cells	Type II target cells
Unabsorbed	1:2,048	1:4,096
Absorbed with type I cells	1:32	1:64
Absorbed with type II cells	1:128	1:256

### Electron microscopy of type I and type II strains

**Figure 6:**
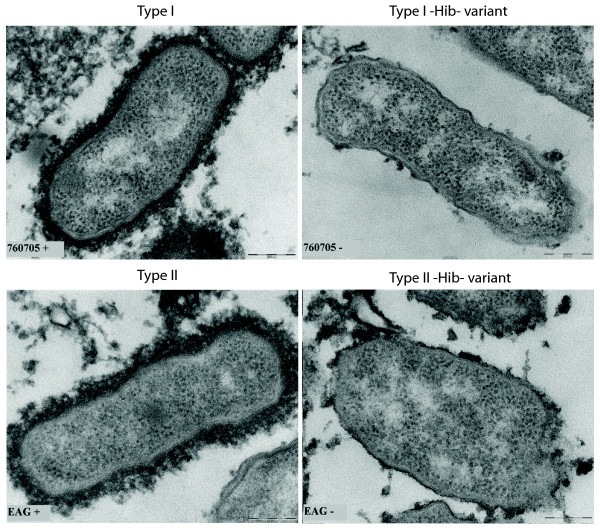
**Comparison of capsular structures of type I and type II Hib cells by electron microscopy**. Overnight cultures were stained for the presence of polysaccharide capsule, fixed, counterstained and cut into ultrathin sections. All images were made at 67,000× magnification.

To determine if the observed difference in capsular expression could also be visualized on the surface of the bacterial cells, electron microscopy (EM) was performed. For this purpose overnight grown type I (H1976-0705) and type II (Eagan) cultures and their isogenic capsule minus variants were stained and fixed to visualize capsular structures on the surface of the cells. Examination by electron microscopy revealed that type I cells had a thinner capsular layer than the cells from type II strain (Fig. 6). The average thickness of the capsular layer of type I cells and type II cells was 42 nm (range 16–104 nm, SE 13 nm) and 72 nm (range 23–152 nm, SE 23 nm), respectively. However, the capsule of type I cells was more electron dense, appearing as a more compact layer on the cell surface compared the capsule of the type II cells.

## Discussion

In this study, we determined the composition of the genes encoding the components required for the synthesis and transport of the polysaccharide capsule of Hib strains isolated from patients with invasive disease in the Netherlands. We found that two variants of the capsular gene cluster exist and we designated these as type I and type II. Type I strains were found to carry approximately twice as much capsular polysaccharide on the surface of the cells as did type II strains. Of the strains, isolated from patients with invasive Hib disease in the Netherlands during the time period 1983–1995, 5% were type II. After 1995, only 2 years after the introduction of the Hib vaccine in the Dutch national immunization program, type II strains were no longer isolated from Dutch patients with invasive Hib disease.

The variation between the capsular gene cluster sequences of type I and type II strains was predominantly found in the *hcsA *and *hcsB *genes. The nucleotide sequences of *bex*, *bcs1 *and *bcs2 *of type I and of type II were identical. Only one SNP was found in *bcs3 *and a few SNPs in *bcs4*, some of which are leading to amino acid substitutions. The *bcs *genes are believed to be involved in the biosynthesis of the polysaccharide and even small alterations in these genes could change the composition of the polysaccharide produced. However, NMR analysis of the polysaccharides isolated from type I and type II strains revealed no structural differences between the two types. Recently, Sukupolvi-Petty and colleagues demonstrated that the products of the *hcsA *and *hcsB *genes facilitate transport of capsular polysaccharide across the outer membrane [7]. Inactivation of *hcsA *alone resulted in accumulation of polysaccharide in the periplasm and a partial decrease in surface-associated polysaccharide, whereas inactivation of *hcsB *alone or of both *hcsA *and *hcsB *resulted in accumulation of polysaccharide in the periplasm and complete loss of surface associated polysaccharide. It is therefore feasible that alterations in the *hcsA *and *hcsB *genes would influence the degree of export of the capsular polysaccharide to the surface of the Hib cells or the efficiency of retaining the exported polysaccharide on the surface. We showed that the type I *hcsA *and *hcsB *genes and putative encoded proteins differed considerably in composition from those of the type II strains. These differences did not lead to a detectable difference in structure between the two capsular genotypes. However, we provided evidence that, at least *in vitro*, the amount of capsular polysaccharide retained on the surface of studied type I strains was twice as high as that in type II strains. In an inhibition ELISA, cells obtained from type I cultures caused significantly more inhibition than cells from type II cultures. Similar results were obtained in an SBA where absorption of serum samples with type I cells resulted in a four-fold higher reduction of SBA titer compared to absorption with type II cells. Furthermore, there always was a pronounced pro-zone effect when type I strains were used in the SBA while only a modest pro-zone effect was seen when type II strains were used as target cells in the SBA. The pro-zone effect is thought to reflect a steric hindrance caused by high concentrations of bound antibodies which in turn is caused by high concentrations of antigen [11]. This would confirm the higher density of capsular polysaccharide on the surface of type I cells. Electron microscopy revealed that the capsular layers of type I Hib cells were thinner, yet denser than that of type II cells, that could suggest that in type I strains more polysaccharide may bound per surface area.

Cerquetti and colleagues showed that a significantly greater proportion of strains isolated from vaccine failures in the United Kingdom carried multiple copies of the *cap *gene cluster [12]. As shown by Noel and colleagues, the presence of multiple copies of the *cap *locus would result in more polysaccharide production and decreased complement-mediated lysis [13]. Hence, higher antibody levels may be required to protect against Hib disease caused by strains with multiple copies of the *cap *gene cluster. We have shown that type I strains may produce more capsular polysaccharide and therefore higher antibody titers may be required to eliminate type I Hib compared to type II strains. All Dutch type II strains used in this study were exclusively isolated from non-vaccinated children younger than 5 years of age and consequently, all vaccine failures in the Netherlands were caused by type I strains. This suggests that type II Hib strains may be less well equipped to cause invasive disease in hosts that have been immunized by vaccination or by natural exposure to Hib and may explain why the type II strains seem to have become extinct in the Netherlands after the introduction of the Hib vaccine. The fact that the proportion of type II strains among the Hib population before the introduction of the vaccine was only 5% possibly would have contributed to the rapid elimination.

The type I and type II strains appear to represent two distinct groups within the Hib population. This is particularly obvious from the MLST analysis of the Hib strains. The Hib population displays a relatively low degree of genetic diversity when analyzed by MLST. In the Netherlands 75% of the Hib strains typed by MLST had ST-6. Remarkably, none of the Dutch type II strains were ST-6, suggesting an inverse relationship between capsular genotype II and ST-6. This inverse relationship was confirmed by our finding that none of the type II strains obtained from other countries had ST-6. MLVA analysis of the Dutch Hib collection also revealed a non-random distribution of the type II strains corroborating the distinct nature of the type II strains.

It is unclear what has caused the polymorphism of the two gene clusters in Hib strains, but it is unlikely that this reflects a gradual change in the capsular genes. DNA sequence analysis of the complete capsular gene clusters of 11 Hib strains revealed the presence of only 2 types of sequences. Sequence analysis of a large number of *hcsA *PCR products obtained from Dutch type I and type II Hib and from strains collected in various other parts of the world confirmed the very limited variability in type I strains and the complete lack thereof in those from type II strains. Possibly Hib strains have acquired the *hcsA *and *hcsB *genes from other capsulated *H. influenzae *or other bacterial species through lateral transfer and recombination. Thus far only the *hcsA *and *hcsB *genes of serotype b and serotype f strains have been sequenced [14]. Comparison of the type I and type II *hcs *gene sequences of the Hib strains with the *hcs *sequences of the serotype f strain shows that these are similar but distinct sequences and that Hib has not acquired the *hcs *genes from *H. influenzae *serotype f.

## Conclusion

In summary, this investigation has shown that two variants of Hib strains exist that differ considerably in the DNA sequence of the genes *hcsA *and *hcsB*. These genes are involved in the transport of the major virulence factor, the capsular polysaccharide, to the surface of the Hib cell. We show that the type I isolates used in this study carried more surface bound capsular polysaccharide than type II isolates. Type II strains were isolated from 0–4 year old, non-vaccinated patients suggesting type II only causes invasive disease in hosts that have not yet mounted antibodies against the Hib capsule. Before the introduction of the Hib vaccine 5% of the Hib isolates from Dutch patients with invasive Hib disease were type II and after nationwide Hib vaccination this type was no longer found. The lower capsule production in type II strains may have caused a selective disadvantage explaining the rapid disappearance of type II strains from the Dutch Hib population. However, this change in the composition of the Hib population does not explain why the Netherlands has experienced an increase in the number of vaccine failures since 2002 and further investigation is required to explain the observed increase.

## Methods

### Bacterial strains and serum samples

For this study we included 670 Hib strains collected during the years 1983–2006 by the Netherlands Reference Laboratory for Bacterial Meningitis (NRBM) from Dutch patients with invasive disease. Of these strains 409 were isolated from CSF and 245 strains were isolated from blood. The remainder of the strains was isolated from other normally sterile compartments such as joints. Hib strains are sent to the NRBM on a voluntary basis, but it is estimated that this results in coverage of more than 85% of all cases of invasive Hib disease in the Netherlands. The strain collection contained all available 282 Hib strains isolated from 1996–2006 and 388 randomly selected strains from the time period 1983–1996. From each year of latter time period, we included approximately 20 Hib strains isolated from 0–4 year old children and approximately 10 strains from patients older than 4 years, 75% of which were 18 years or older. In addition, we used Hib reference strain Eagan and vaccine strain 1482 kindly provided by Dr. R. Schneerson. Dr. L. van Alphen kindly provided us with 134 Hib strains from different locations in the world collected during the years 1980–1990. However, the nature of these strains, carriage or invasive disease, was unknown. Of the Dutch Hib strains 667 were typed by multiple-locus variable number tandem repeats analysis (MLVA) [9] and 237 by multi-locus sequence typing (MLST) [10,9].

Positive serum samples for SBA and ELISA were obtained from 5 volunteers who received a single dose of ActHib vaccine (Aventis Pasteur MSD, Lyon, France) and a negative control serum was obtained from a volunteer who was not immunized (ages ranged from 25 to 57 years). Serum samples were collected 2 months after vaccination, aliquoted and stored at -20°C until use.

### DNA sequencing of the capsular gene cluster

For sequencing of the complete Hib capsule gene cluster PCR products creating overlapping fragments of the gene cluster were used. Sequence reactions were performed with the ABI PRISM BigDye Terminator cycle sequencing kit v3.1 (Applied Biosystems, Foster City, Calif.) and analyzed on an AB3700 DNA sequencer. DNA sequences have been submitted to GenBank under acc.no. DQ368334 and acc.no. DQ368335.

### Hib capsular genotype specific PCR

For PCR detection of the type I *hcsA *oligonucleotide primers HiHcsA12667F-I (GTACTTGTCATTGACCAAACTTT) and HiHcsA13116R-I (GGTATATTGAAAGTATGCTGCAT) yielding a 450 bp PCR product were used. To detect type II *hcsA *primers HiHcsA12668F-II (TGCTTGTCATCGATCAAA) and HiHcsA13484R-II (ACTAAAGAAAGGGGTGCAA) yielding a 817 bp PCR product were used. Two separate PCRs were performed in AB9700 PCR machines using the following protocol: 1 μl of 1:10 diluted heat-treated *H. influenzae *lysate was added to a 24 μl mixture containing 10 pmol of each primer and 12.5 μl diluted HotStar Taq mastermix (Qiagen, Hilden, Germany). The PCR program used was 15 min at 95°C, followed by 30 cycles of amplification that consisted of 30 sec at 95°C, 1 min at 52°C, and 1 min at 72°C and a final 7 min at 72°C. Strains were screened by type I PCR and all samples that did not yield a PCR product were analyzed again in both the type I and type II PCR. All strains for which the *hcsA *PCR products were used for sequence analysis were analyzed in both PCRs.

### Serum bactericidal activity

Functional antibodies binding to the PRP capsule of Hib and fixing complement onto the bacterial surface were measured by an slightly modified assay for serum bactericidal activity (SBA) described by Romero-Steiner et al. [15]. Ten μl of heat inactivated serum was mixed with 20 μl of the diluted Hib suspension and incubated for 15 min at 35°C at 5% CO_2_. Thereafter, 25 μl of Hanks buffer (Life Technologies, Grand Island, N.Y., USA) with 2% Fildes (BBL, Becton Dickinson and Co., Sparks, Md., USA) and 25 μl of baby rabbit complement (Pel-Freez, Brown Deer, Wis., USA) was added and the mixture was incubated for 1 h at 35°C at 5% CO_2_. From the mixture 5 μl aliquots were applied onto 12 × 12 cm square culture plates filled with Brain Hearth Infusion agar, supplemented with Haemophilus test medium supplement (Oxoid, Haarlem, The Netherlands). SBA titers were defined as the serum dilution that resulted in >90% killing of the Hib culture used for the assay.

### Inhibition of the serum bactericidal activity

Hib suspensions used for absorption assays were obtained from 100 ml overnight cultures, grown in supplemented Brain Hearth Infusion broth. Fifty ml of culture was centrifuged for 10 min at 3000 g, and the pellet was resuspended in 5 ml 150 Mm NaCl after which the bacteria were killed by a 10 min incubation at 65°C. In the SBA absorption assays 10 μl aliquots of heat inactivated serum samples were pre-absorbed with 10 μl heat killed Hib suspensions. The mixture was incubated for 1 h at 37°C followed by an overnight incubation at 4°C. The mixture was then centrifuged for 10 min at 10,000 g and the supernatant was used for SBA. To ensure the various cultures contained the same number of bacteria, real time quantative PCR and protein quantification were used to assess and adjust the number of bacteria in each culture used for absorption. Cell suspensions and supernatants were aliquoted and stored at -20°C until use.

### Type b polysaccharide inhibition ELISA

To assess the level of the capsular polysaccharide expression of the various Hib strains an inhibition ELISA was designed. For this purpose an ELISA, described by Phipps et al. [16] and used to determine antibody titers against Hib polysaccharide was adapted. In contrast to the original method 1:20,000 diluted horse-radish-peroxidase labeled Protein A/G (Pierce, Rockford, USA) and TMB substrate solution (110 mM NaAc pH 5.5, 166 μg/ml 3,3',5,5' tetramethylbenzidine, 0.006% H_2_O_2_) were used. Reactions were stopped after 5 min using 2 M H_2_SO_4_, and values were read at 450 nm in a Biotek EL312e ELISA reader. In order to relate the degree of inhibition to the amount polysaccharide, serial dilutions of purified polysaccharide (HBO-HA, National Institute for Biological Standards and Control, Hertfordshire, United kingdom) with known concentration were added to HBO-HA-coated microtiter plates after which a single dilution of a high titer serum sample was added to all wells and incubated for 1 h at room temperature. Subsequently the ELISA was performed as described above.

Hib suspensions used for the inhibition ELISA were obtained from the same overnight grown type I and type II cultures as those used for the absorption of the SBA. Five ml culture was centrifuged as described above and the pellet was reconstituted in 5 ml PBS after which Hib in both supernatant and cellular fraction were heat killed. To ensure the various cultures contained the same number of bacteria, real time quantative PCR and protein quantification were used to assess and adjust the number of bacteria in each culture used for absorption. To assess the polysaccharide content in Hib strains, the above described cells and culture supernatants from type I and type II strains were pre-treated with 0.1% SDS for 30 min at 60°C and used in the inhibition ELISA described above.

### Electron microscopy of Hib strains

Fixation and capsular stain were basically performed according to the lysine-acetate-based formaldehyde-glutaraldehyde ruthenium red-osmium fixation procedure (LLR-methode) method [17]. A 30 ml overnight culture of Hib grown in supplemented BHI was centrifuged for 10 min at 1800 g. Half of the supernatant was discarded and the remaining cells were fixed in paraformaldehyde and glutaraldehyde in the presence of ruthenium red after which they were imbedded in agarose by centrifugation, dehydrated using graded series of ethanol, impregnated in the epoxy resin glycidether 100 and finally polymerized in BEEM capsules at 60°C. Ultrathin sections were cut with a diamond knife, contrasted with 2% uranyl acetate, counterstained with lead citrate and examined in a FEI Tecnai12 transmission electron microscope.

### Purification of the Hib polysaccharide

The capsular polysaccharide of various Hib strains was isolated and purified from liquid cultures as described before with some modifications [18]. Strains were cultured 22 hours at 35°C in 500 ml supplemented brain heart infusion broth. The culture was centrifuged for 30 min at 3000 g and 0.65% cetyl trimethyl ammonium bromide (CTAB) was added to the culture supernatant. After overnight incubation at 4°C the solution was centrifuged for 30 min. at 3000 g. The pellet was dissolved in 50 ml solution containing 1 M NaCl, 5 mM NaAc pH5.2 and 72% ethanol was added. After overnight incubation the solution was centrifuged for 30 min at 3000 g and the pellet was dissolved in 10 ml PBS (200 mM NaCl, 4.5 mM KCl, 17 mM phosphate buffer pH7.4). The solution was then purified on a Hi Prep 16/60 Sephacryl S300 high resolution column (GE Healthcare, Uppsala, Sweden) using PBS at a flow rate of 0.5 ml/min. The purified polysaccharide was eluted from the column in a 12 ml volume directly after the void volume. The purified polysaccharide was precipitated by adding 1 M NaCl, 5 mM NaAc pH5.2 and 72% ethanol and overnight incubation at 4°C. After centrifugation for 30 min. at 3000 g the pellet was dissolved in water, dialyzed extensively against water in a slide-a-lyzer with a 7000 Da cut-off (Pierce Biotechnology, Rockford, USA) and dried in a SpeedVac. The average yield of this procedure was 5–10 mg of purified PRP.

### Nuclear magnetic resonance analysis of Hib polysaccharide

The purified PRP was analyzed by nuclear magnetic resonance (NMR) as described before [19]. Briefly, samples were dissolved in deuterated water (D2O), containing 0.075% (w/w) of trimethylsilyl-[D4]-propanoate sodium salt (Sigma-Aldrich, Zwijndrecht, The Netherlands). NMR-spectra were recorded on a JEOL JNM-ECP400 FT NMR system (JEOL, Tokyo, Japan) at 9.4 T using a pre-saturation pulse to lower the residual water peak.

## Authors' contributions

CS sequenced the capsular gene clusters and performed the type I and II PCRs. HH and CS performed the analysis of the polysaccharide expression. SW did most of the MLST and MLVA. BZ performed the NMR. MB performed the electron microscopy. AE collected and provided the bacterial strains. LS initiated and managed the project and wrote the manuscript. All authors approved the final manuscript.
